# Decision tree–based identification of *Staphylococcus aureus* via infrared spectral analysis of ambient gas

**DOI:** 10.1007/s00216-021-03729-2

**Published:** 2021-10-23

**Authors:** Hidehiko Honda, Masato Yamamoto, Satoru Arata, Hirokazu Kobayashi, Masahiro Inagaki

**Affiliations:** Faculty of Arts and Sciences at Fujiyoshida, Showa University, 4562, Kami-yoshida, Fuji-yoshida-shi, Yamanashi 403-0005 Japan

**Keywords:** Bacteria identification, Decision tree, Infrared absorption spectra, Machine learning, *Staphylococcus aureus*

## Abstract

In this study, eight types of bacteria were cultivated, including *Staphylococcus aureus*. The infrared absorption spectra of the gas surrounding cultured bacteria were recorded at a resolution of 0.5 cm^−1^ over the wavenumber range of 7500–500 cm^−1^. From these spectra, we searched for the infrared wavenumbers at which characteristic absorptions of the gas surrounding *Staphylococcus aureus* could be measured. This paper reports two wavenumber regions, 6516–6506 cm^−1^ and 2166–2158 cm^−1^. A decision tree–based machine learning algorithm was used to search for these wavenumber regions. The peak intensity or the absorbance difference was calculated for each region, and the ratio between them was obtained. When these ratios were used as training data, decision trees were created to classify the gas surrounding *Staphylococcus aureus* and the gas surrounding other bacteria into different groups. These decision trees show the potential effectiveness of using absorbance measurement at two wavenumber regions in finding *Staphylococcus aureus*.

**Graphical abstract:**

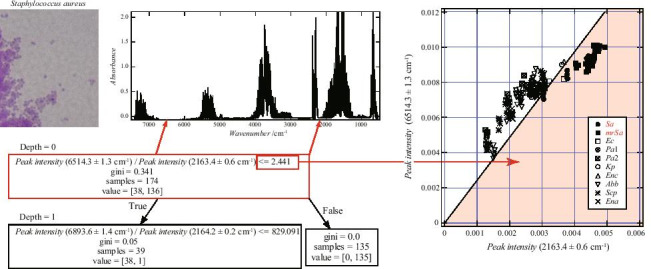

## Introduction

The presence of the *Staphylococcus* (*S.*) *aureus* species in humans is associated with the occurrence of several health hazards, including bacteremia, infective endocarditis, osteomyelitis, pulmonary infections, gastroenteritis, meningitis, toxic shock syndrome, and urinary tract infections [1–4]. Annually, the USA alone records up to 20,000 deaths, which are partly attributable to the presence of *S. aureus* [5]. Accordingly, precise, effective prevention and treatment methods must be developed to neutralize the effects of such lethal bacteria. From a medical research perspective, this entails the development of effective procedures for monitoring the strain and reproduction rate of *S. aureus*.

Infrared laser radiations and optical parametric generations that oscillate at a specific wavenumber have been proposed as a means of facilitating easy and continuous analysis of the volatile organic compounds contained in human exhaled breath [6–8]. If the presence of bacteria could be confirmed by laser spectroscopy, reproduction monitoring could be realized by measuring the surrounding air without establishing contact with the concerned bacteria. However, this method has not been applied to the reproduction monitoring of *S. aureus.* A similar method to analyze the reproduction of *S. aureus* would facilitate the realization of healthy human life, but appropriate investigations in this regard are yet to be undertaken. This study aims to address this gap in the existing literature.

Bacterial species possess unique characteristic odors. These odors arise because of the release of volatile organic compounds characteristic of each species into the atmosphere. Previous studies have utilized gas chromatography and mass spectrometry to demonstrate that the *S. aureus* metabolites contain isovaleric acid and 2-methyl-butanal [9]. However, infrared spectroscopy fails to detect these volatile metabolites because their derived peak does not always appear solely at the position of the strong infrared absorption peak obtained from their stable molecular structure [10]. This might result in the aggregation of several characteristic molecules that absorb infrared rays. Furthermore, any overlap with the absorption peaks of other molecules can potentially impede metabolite quantification [11]. This necessitates the availability of the characteristic-spectrum wavenumber information to realize accurate metabolite detection via infrared spectroscopy [12, 13].

The proposed research employs a decision tree–based machine learning algorithm to detect the wavenumber range across which the infrared absorption spectra peculiar to *S. aureus* can be recorded. Recent advances in machine learning technology have demonstrated its significant potential with regard to efficiently handling classification tasks involving large quantities of data that cannot be analyzed by humans; moreover, it can handle data that are too small in value to be distinguished using ordinary analysis methods [14–20]. This paper presents an approach to classify infrared absorption spectra via machine learning. A dataset comprising approximately 5 × 10^9^ data points was used to this end. The results obtained in this study demonstrate the realization of accurate bacterium-propagation detection in the gas surrounding *S. aureus* via infrared irradiation of the odorous surrounding space.

## Materials and methods

### Sample collection

**Fig. 1:**
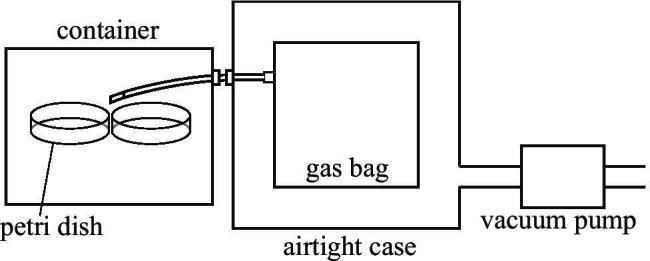
Collection of mixed gas. Petri dishes inoculated with bacteria were placed in a closed container and placed in an incubator. After the culture time shown in Table 1 had elapsed, a silicone tube was inserted through the insertion port of the closed container. Subsequently, the gas around the Petri dish was recovered in the gas bag, which was inside an airtight case that gave the gas bag a negative pressure via a vacuum pump

Eight common types of bacteria were cultivated in this study. Table 1 lists these bacteria types considered along with their specifications. The *S. aureus* species considered in this study include the standard type (Id: *Sa*) and its drug-resistant strain (*mrSa*). All bacteria types were cultivated in sheep-blood medium consisting of casein peptone 13.0 g L^−1^, soybean protein digest 5.0 g L^−1^, growth factors 2.2 g L^−1^, NaCl 5.0 g L^−1^, agar 13.0 g L^−1^, and 5% defibrinated sheep blood (Nissui Plate Sheep Blood Agar 51,001, Nissui Pharmaceutical Co., Ltd.). Only one type of bacteria was planted in each Petri dish. Two Petri dishes planted with the same type of bacteria were placed in an airtight container, and the bacteria were cultured in an incubator while inside the airtight container. The culturing was performed at a temperature of 37 °C over a period of approximately 40 h. Postculturing, the gas surrounding the bacterium was aspirated into gas bags (smart bag PA, smart bag 2F, Tedlar bag or ANALYTIC-BARRIER Bag, GL Sciences) by the indirect sampling method using a dry pump, as shown in Fig. 1. Each gas bag was equipped with a standard sleeve (6–7 mm O.D.) connected to a silicone tube within 1 m. The other end of the silicone tube was placed within 5 cm of the object.

**Table 1:** Eight facultative anaerobic bacteria species cultured in this study in sheep-blood medium. *Staphylococcus aureus* and *Pseudomonas aeruginosa* were cultivated in two types, a standard bacterium and a drug-resistant bacterium. For the other six species of bacteria, either standard bacteria or drug-resistant bacteria were used as samples. “Number of samples” is equal to the number of cultures. For example, *methicillin-resistant Staphylococcus aureus* (ID: *mrSa*) was cultivated seven times, and a sample of the gas around the bacterium was collected in each culture. In the case of *mrSa*, the absorbance was measured once to four times for each sample. The number in parentheses in “Total number of measurements” is the number of absorption spectra measurements for each sample, and the total number of infrared absorption spectra (Total number of measurements) is 1 + 1 + 4 + 4 + 4 + 4 + 4 = 22 spectra

Air around bacteria	Genus species	ID	Strain/origin	Number of samples	Total number of measurements	Incubation time (h)
Standard strains	*Staphylococcus aureus*	*Sa*	Type strain (NBRC 13,276)	4	16 (4 + 4 + 4 + 4)	39
Hospital isolates	*Methicillin-resistant Staphylococcus aureus*	*mrSa*	Clinical isolate	7	22 (1 + 1 + 4 + 4 + 4 + 4 + 4)	44
Standard strains	*Escherichia coli*	*Ec*	Type strain (NBRC 3972)	2	8 (1 + 7)	32
	*Pseudomonas aeruginosa*	*Pa*1	Type strain (NBRC113275)	2	11 (5 + 6)	38.5
Hospital isolates	*Pseudomonas aeruginosa*	*Pa*2	Hospital environment	4	20 (5 + 5 + 5 + 5)	39
	*Klebsiella pneumoniae*	*Kp*	Clinical isolate	2	8 (1 + 7)	32
	*Enterobacter cloacae*	*Enc*	Clinical isolate	2	8 (1 + 7)	39
	*Acinetobacter baumannii*	*Abb*	Clinical isolate	10	53 (5 + 6 + 5 + 5 + 5 + 5 + 5 + 5 + 6 + 6)	38.5
	*Streptococcus pneumoniae*	*Scp*	Clinical isolate	4	18 (4 + 6 + 4 + 4)	37.5
	*Enterobacter aerogenes*	*Ena*	Clinical isolate	2	10 (4 + 6)	37.5

The number of cultures is shown in the “Number of samples” column in Table 1. One gas sample was obtained from one airtight container. Absorbance measurements were performed multiple times for each sample. The number of infrared absorption spectra obtained for each sample is shown in parentheses in the “Total number of measurements” column. For example, *S. aureus* was cultured four times, each gas sample obtained in each culture was measured four times, and a total of 16 spectra were obtained.

### Methods

#### Infrared spectroscopy

**Fig. 2:**
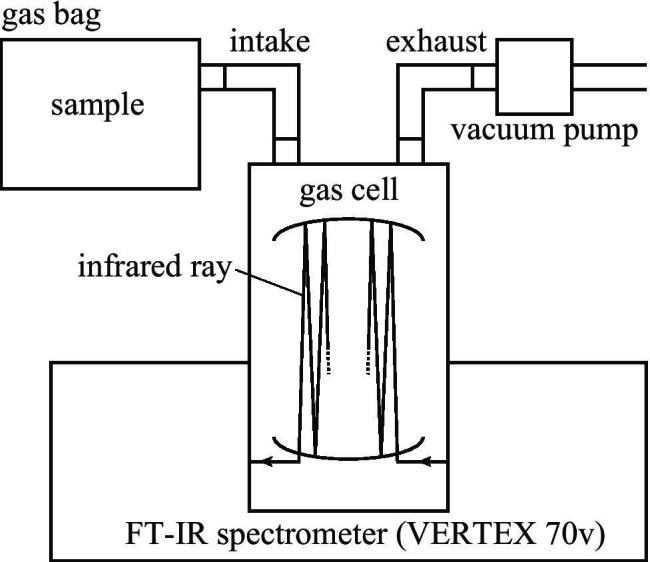
Infrared absorption spectrum measurement. A vertical gas cell was installed in the sample compartment of the infrared spectrophotometer (VERTEX 70v). At the top of the gas cell were an intake and an exhaust. A gas bag was connected to the intake port, and a vacuum pump was connected to the exhaust port. Infrared rays were reflected multiple times in the gas cell as illustrated by the line with arrows

A gas cell characterized by a 10 m optical-path length was set in the sample compartment of the VERTEX 70v FT-IR spectrometer (Bruker Corporation; USA), as shown in Fig. 2. There was an intake port and an exhaust port at the top of the gas cell. The air in the cell was exhausted from the exhaust port with the PM28309-950.50 vacuum pump (KNF Neuberger GmbH; Germany), and background measurement was performed in a vacuum state. Subsequently, by closing the exhaust port and opening the intake port, the sample was introduced into the gas cell by suction until the pressure in the cell reached atmospheric pressure. The infrared absorption spectrum corresponding to the gas was recorded under atmospheric pressure. The measured wavenumber range was 500–7500 cm^−1^, while the corresponding spectral resolution and integration time were 0.5 cm^−1^ and 10 min, respectively.

#### Machine learning

A decision tree algorithm was used to detect the absorption peaks specific to *Staphylococcus aureus* from the infrared absorption spectrum group of the gas around the bacteria. The *classification and regression tree* was used as the decision tree algorithm, and Scikit-learn was used as the library [21]. The details concerning the decision tree algorithm parameters are listed in Table 2. Training data were created by the following three methods. The learning was conducted 150 times, and the calculation was repeated under the condition that the data used to create one decision tree would not be used again. The learner was trained with data for all peak intensity ratios or all ratios of absorbance differences. Therefore, a combination of very small peak intensities or values of very small absorbance differences were sometimes selected. Slight differences in absorbance cannot be measured without using a device with a high signal-to-noise absorbance ratio. To increase the versatility of the results, the decision tree with a relatively high peak intensity and which was judged to be useful was selected from the 150 decision trees; the results are discussed in the following section. Test data were not used in this study as our intention was to classify the training data. We used Microsoft Excel 2016 and 2019 and Visual Studio 2019 to create the training data. For machine learning, we used RAPIDS Docker (21.08-cuda11.0-runtime-ubuntu18.04-py3.8) and Scikit-learn (version 0.23.1).

**Table 2:** Decision tree parameters. Arguments specified by fit function of scikit-learn

Parameter	Value
ccp_alpha	0
criterion	Gimi
splitter	Best
class_weight	None

##### Classification by peak intensity ratio

Numerous peaks were observed in the infrared absorption spectra obtained for each bacteria type. The wavenumber and absorbance values as well as minimum values on both sides of the absorption peaks were extracted. The points corresponding to the minimum absorbance values were considered to constitute the baseline. The difference between the peak and baseline absorbance values was calculated and used as the peak intensity. A maximum of 7667 peak intensities were detected for each spectrum. The absolute peak intensity value was proportional to the sample gas concentration, i.e., the partial pressure of gas released by the bacteria. Consequently, the intensity values differed from one gas sample to another. To eliminate the influence of sample-gas concentration, the ratio of any two peak intensities was used to classify the infrared absorption spectra. In other words, the infrared absorption spectra were characterized using the mixing ratio of the two partial structures that absorbed infrared rays. The ratio of all peak intensities to other peaks was calculated and considered as learning data. The peak intensity ratio data were a maximum of 2.94 × 10^7^ real array. Because this study used 174 infrared absorption spectra, the training data had a structure of 2.94 × 10^7^ columns × 174 rows. Each row of the training data was labeled (i) “gas surrounding *S. aureus* (*Sa* and *mrSa*)” and (ii) “others”—comprising 38 and 136 infrared absorption spectra, respectively.

##### Classification based on absorbance difference at two wavenumbers

When generating training data in Method 1, the peak intensity—that is, the difference between the peak and baseline absorbance values—was determined from the infrared absorption spectra. Therefore, when calculating the peak intensity, it is necessary to determine the peak absorbance value along with the corresponding minimum values on both sides of the peak. The wavenumbers that determine the absorbance values at three points differ across spectra. Therefore, to calculate the peak intensity, it is necessary to measure the absorbance by changing the wavenumber in the wavenumber range where the peak is detected. When measuring infrared absorption outside laboratory settings and classifying the infrared absorption spectra of mixed gases on the spot, it is desirable to fix the wavenumber when measuring the absorbance; for this, an infrared-light-emitting diode can be used.

Considering this application, we also formulated a procedure for classifying the difference between absorbance values at two points with constant wavenumbers. In this method, training data were created using the absorbance values in the wavenumber region where the peak specific to *S. aureus* was detected, which was found in Method 1. The wavenumber regions were 6515.6–6507.6 cm^−1^ and 2158.4–2166.3 cm^−1^. Each region contained 67 absorbance data. The difference between each absorbance value and another absorbance value was calculated for each of the two regions. The difference in absorbance was an array of 2211 values in each region. We calculated the ratio of the values in both arrays and created an array of 2211 × 2211 = 4.89 × 10^6^ values. Subsequently, as in Method 1, the arrays corresponding to each of the 174 spectra were arranged in the row direction to be training data, and classes (i) and (ii) labels were assigned.

##### Classification performed using three absorbance values

The absorbance values measured at three points within each wavenumber domain (six points in total) were used to classify the infrared absorption spectra. This approach is similar to that considering peak intensity. However, the wavenumbers corresponding to the absorbance values used in this calculation were maintained constant. This method used the difference in the shape of the spectrum to classify the spectra. Similar to Method 2, the absorbance values at three points were selected for the wavenumber region where the peak intensity was detected. We calculated a straight line connecting the point with the lowest wavenumber and the point with the highest wavenumber. A vertical line was drawn from the remaining one point to a straight line, and the difference in absorbance was calculated along the vertical line. Training data were created by replacing the ratio of the difference values on the vertical line with the ratio of the difference in absorbance used in Method 2.

## Results and discussions

### Classification by peak intensity ratio

**Fig. 3:**
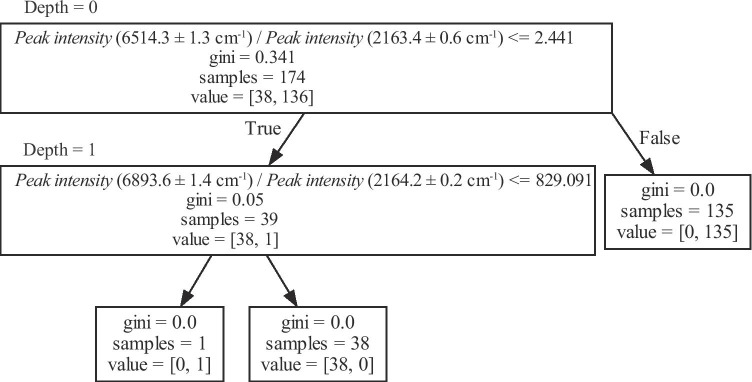
Decision tree generated by Method 1 (using peak intensity ratio as training data). The spectrum of *S. aureus*, *Sa* and *mrSA* (38 spectra), and the spectrum of other bacteria (136 spectra) were separated. The numbers in parentheses are the wavenumber ranges that include the peaks. At Depth 0, the spectra were divided into two groups depending on whether the peak intensity ratio was less than or exceeded 2.441. All *Staphylococcus aureus* spectra were classified in the group with a peak intensity ratio of 2.441 or less, and only one spectrum of other bacteria was included in this group

Figure 3 illustrates the decision tree generated by Method 1. The spectral separation was performed in two stages—“Depths 0–1.” The training data comprised 174 spectra, including two classes—peripheral gases obtained by culturing (i) *S. aureus* and (ii) other bacteria, containing 136 and 38 spectra, respectively. During Depth 0 classification, 99% separation was achieved, and the spectra were classified based on the ratio of peak intensities corresponding to the (6514.3 ± 1.3) and (2163.4 ± 0.6) cm^−1^ wavenumbers. The 174 spectra at Depth 0 were divided into two groups comprising 38 + 1 and 135 spectra, depending on whether the above-described peak ratio was less than or exceeded 2.441. The former group comprised 38 and 1 spectra corresponding to classes (i) and (ii), respectively. Accordingly, these 38 + 1 spectra were divided into two classes referred to as “Depth 1.”

**Fig. 4:**
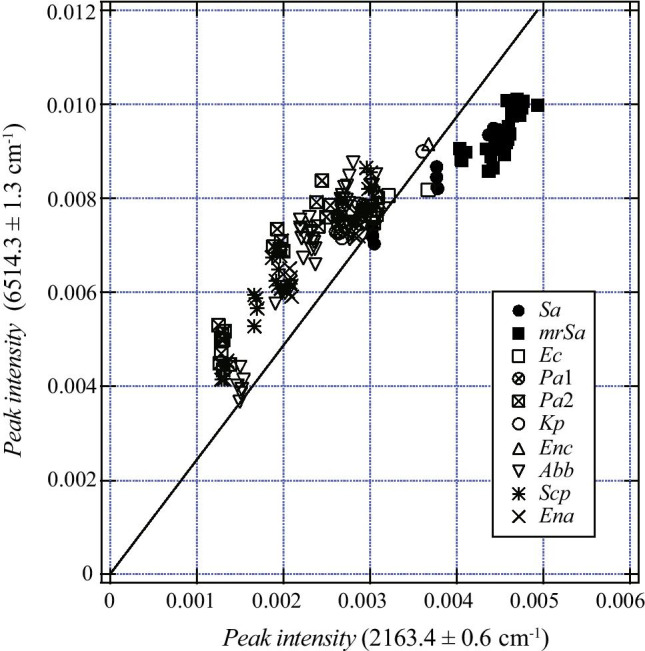
Peak intensity data used to generate the decision tree shown in Fig. 3. The vertical axis is the peak intensity value in the range of 6514.3 ± 1.3 cm^−1^, and the horizontal axis is the peak intensity value in the range of 2163.4 ± 0.6 cm^−1^. The dark black markers indicate results obtained for the gas surrounding *S. aureus*, and other marks are data for bacteria other than *S. aureus*. The slope of the straight line passing through each point and the origin is the peak intensity ratio. The slope of the solid line is 2.441, which is the boundary value obtained by the decision tree. All *S. aureus* data are plotted in the area to the right of the border. There is one □ plot in this area. This is *Escherichia coli* data

Figure 4 depicts the peak intensity distribution observed in the (6514.3 ± 1.3) and (2163.4 ± 0.6) cm^−1^ wavenumber ranges. The dark black markers indicate values obtained from the gas surrounding *S. aureus*. The gradient of the straight line depicted in the figure is 2.441, and it corresponds to the value on the right side of the classification relation for Depth 0 (Fig. 3). Most spectra with peak intensity ratios of 2.441 or less were included in the class containing *S. aureus*.

**Fig. 5:**
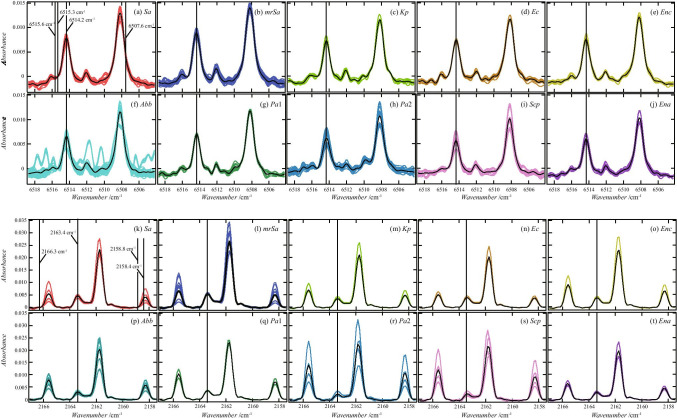
Absorbance from 6518.7 to 6504.2 cm^−1^ (**a**–**j**) and that from 2167.0 to 2157.5 cm^−1^ (**k**–**f**). All 174 spectra used in this study are plotted. The colored curves are the spectrum of the gas around each bacterium, and the solid black curves are the curves obtained by averaging the absorbance. These wavenumber regions (from 6518.7 to 6504.2 cm^−1^, from 2167.0 to 2157.5 cm^−1^) include the regions (6514.3 ± 1.3 cm^−1^ and 2163.4 ± 0.6 cm^−1^) where the peaks selected by the decision tree shown in Fig. 3 were observed. The absorbances measured from 6515.6 to 6507.6 cm^−1^ and from 2158.4 to 2166.3 cm^−1^ were used when creating training data in Methods 2 and 3. Vertical axis values were adjusted to ensure the absorbance at 6515.3 and 2158.8 cm^−1^ equals zero

Figure 5 shows the spectrum at approximately 6514.3 and 2163.4 cm^−1^. All spectra were overlaid for each bacteria type. The black lines are the average curves of absorbance. (Hereinafter, the curves are used as curves representing the characteristics of the spectral shape for easy viewing.) Table 3 shows the values of the mean and the standard deviation of the absorbance at 6514.2 cm^−1^ and 2163.4 cm^−1^.

**Table 3:** Average and standard deviation of the absorbance of 6514.2 cm^−1^ and 2163.4 cm^−1^ of the gas around the bacteria. The average values are the points on the solid curves in Fig. 5

ID		*Sa*	*mrSa*	*Kp*	*Ec*	*Enc*	*Abb*	*Pa*1	*Pa*2	*Scp*	*Ena*
6514.2 cm^−1^	Average	0.007842	0.008933	0.007277	0.007461	0.007611	0.006627	0.007007	0.006105	0.005676	0.006046
	Standard deviation	0.000735	0.000576	0.000506	0.000282	0.000536	0.001085	0.000206	0.001330	0.001230	0.000807
2163.4 cm^−1^	Average	0.004632	0.005409	0.003459	0.003905	0.003808	0.002959	0.003493	0.001986	0.002133	0.002878
	Standard deviation	0.000602	0.000292	0.000370	0.000272	0.000222	0.000531	0.000107	0.000722	0.000789	0.000410

### Classification based on absorbance difference at two wavenumbers

**Fig. 6:**
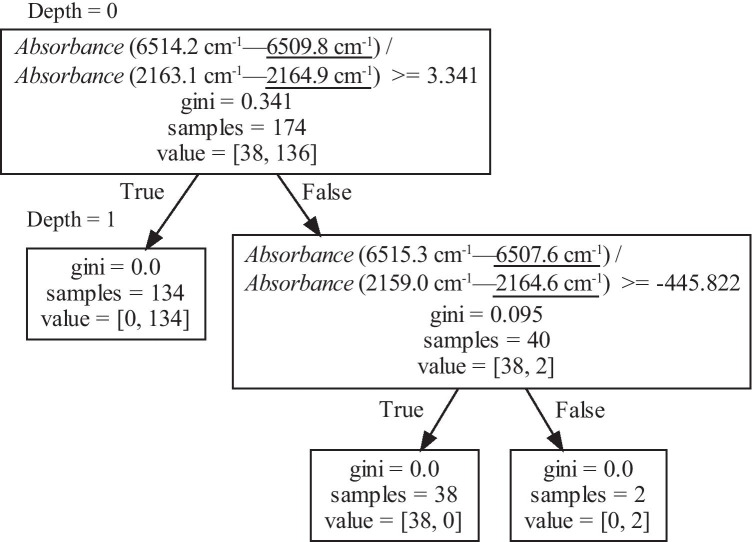
Decision tree generated by Method 2 (using the ratio of the difference in absorbance at two points as training data). “Absorbance (6514.2 cm^−1^—6509.8 cm^−1^)” shows the absorbance at 6514.2 cm^−1^ minus the absorbance at 6509.8 cm^−1^

Figure 6 shows a decision tree generated using the ratio of the absorbance differences as training data. The value selected at Depth 0 was

1 $$\frac{Absorbance\;\mathrm{at}\;6514.2\;\mathrm{cm}^{-1}-Absorbance\;\mathrm{at}\;6509.8\;\mathrm{cm}^{-1}}{Absorbance\;\mathrm{at}\;2163.1\;\mathrm{cm}^{-1}-Absorbance\;\mathrm{at}\;2164.9\;\mathrm{cm}^{-1}}$$

**Fig. 7:**
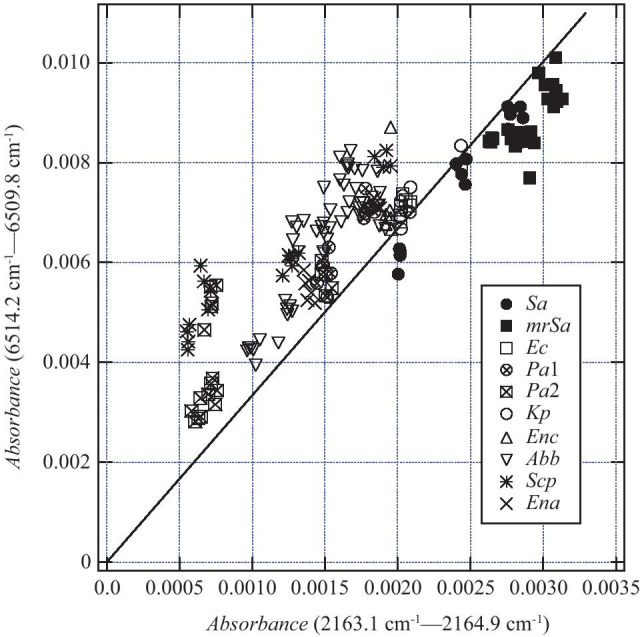
Difference in absorbance used to create the decision tree shown in Fig. 6. The slope of the solid line is the boundary value shown in Fig. 6, 3.341. The slope of the straight line connecting the origin and each point is the ratio of the difference in absorbance

This value was divided into two groups with a threshold of 3.341. Thirty-eight spectra of class (i) and two spectra of class (ii) were mixed in the group with the value less than 3.341, but any spectra other than the two spectra belonging to class (ii) were classified according to the label. Figure 7 shows the distribution of the absorbance difference. The slope of the straight line is 3.341, which is the threshold at Depth 0.

**Fig. 8:**
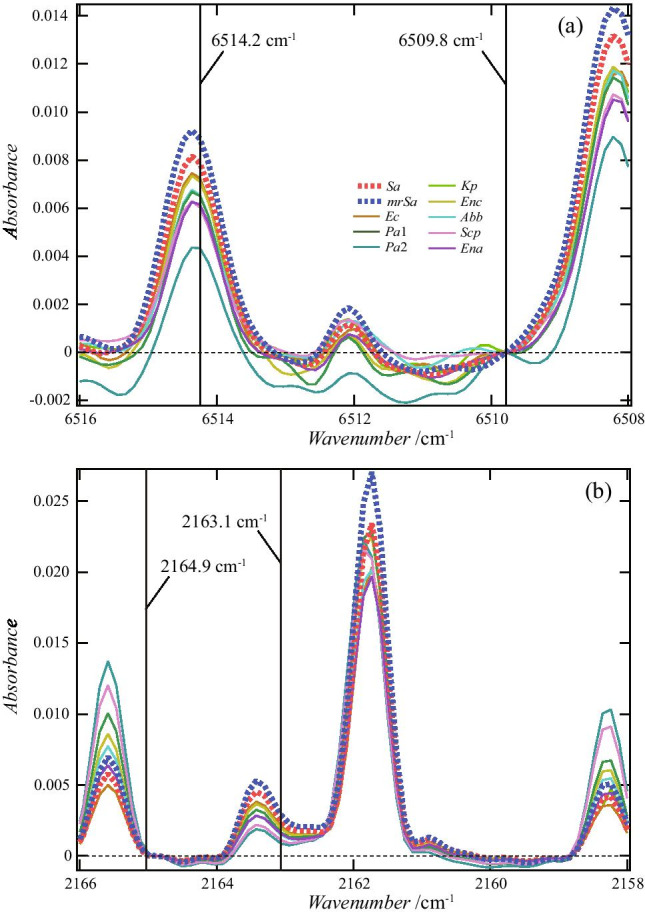
Relationship between 6514.2, 6509.8, 2163.1, and 2164.9 cm^−1^ selected in the decision tree. The absorbance at 6509.8 cm^−1^ and 2164.9 cm^−1^ was plotted to be zero. Therefore, the values on the vertical axis at 6514.2 cm^−1^ and 2163.1 cm^−1^ correspond to the values of the difference in absorbance

Figure 8 shows the four wavenumbers (6514.2, 6509.8, 2163.1, and 2164.9 cm^−1^) extracted by machine learning. The spectra in this figure are curves of the average value of absorbance shown by the black line in Fig. 5. In Figs. 8a and b, the absorbance values are translated so that the absorbances of 6509.8 cm^−1^ and 2164.9 cm^−1^ are zero. Therefore, the value in Eq. (1) is the ratio of the value on the vertical axis of 6514.2 cm^−1^ to the value on the vertical axis of 2163.1 cm^−1^.

### Classification performed using three absorbance values

**Fig. 9:**
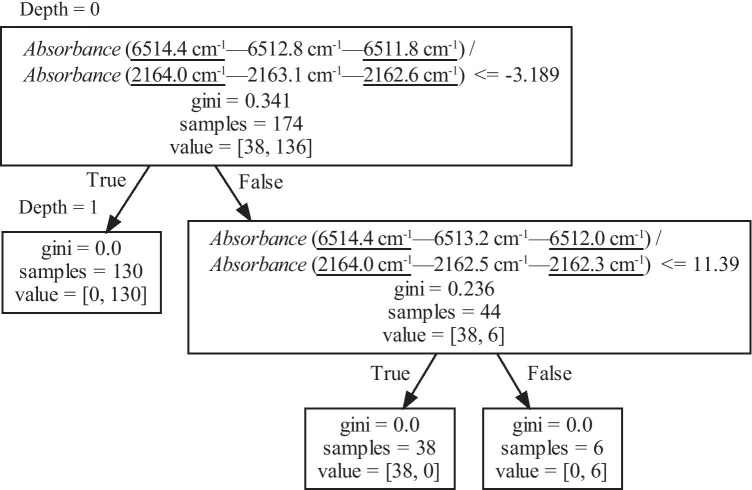
Decision tree generated by Method 3 (method of creating training data using the 3 values of absorbance). “Absorbance (6514.4 cm^−1^—6512.8 cm^−1^—6511.8 cm^−1^)” is the difference between the absorbance obtained at the wavenumber marked in the center, 6512.8 cm^−1^, and the line connected by the two points measured at the underlined wavenumbers, 6514.4 and 6511.8 cm^−1^

Figure 9 shows a decision tree created using the absorbance at three points for each region. The wavenumbers selected by machine learning were replaced as follows:

2 $$\begin{array}{l}w_1=6514.4\;\mathrm{cm}^{-1},\;w_2=6512.8\;\mathrm{cm}^{-1},\;w_3=6511.8\;\mathrm{cm}^{-1},\\w_4=2164.0\;\mathrm{cm}^{-1},\;w_5=2163.1\;\mathrm{cm}^{-1},\;w_6=2162.6\;\mathrm{cm}^{-1}\end{array}$$

The index selected by the machine to classify the spectra was the ratio of

3 $$Absorbance\;\mathrm{at}\;w_2-\left\{\frac{Absorbance\;\mathrm{at}\;w_3-Absorbance\;\mathrm{at}\;w_1}{w_3-w_1}\left(w_2-w_1\right)-Absorbance\;\mathrm{at}\;w_1\right\}$$

to

4 $$Absorbance\;\mathrm{at}\;w_5-\left\{\frac{Absorbance\;\mathrm{at}\;w_6-Absorbance\;\mathrm{at}\;w_4}{w_6-w_4}\left(w_5-w_4\right)-Absorbance\;\mathrm{at}\;w_4\right\}$$

**Fig. 10:**
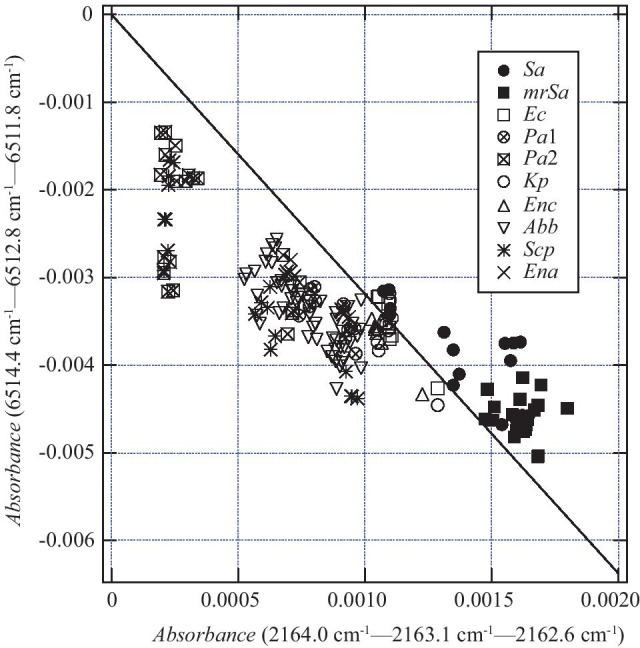
Data of absorbance difference used when creating the decision tree shown in Fig. 9. The slope of the solid line is − 3.189, which is the boundary value at Depth 0

**Fig. 11:**
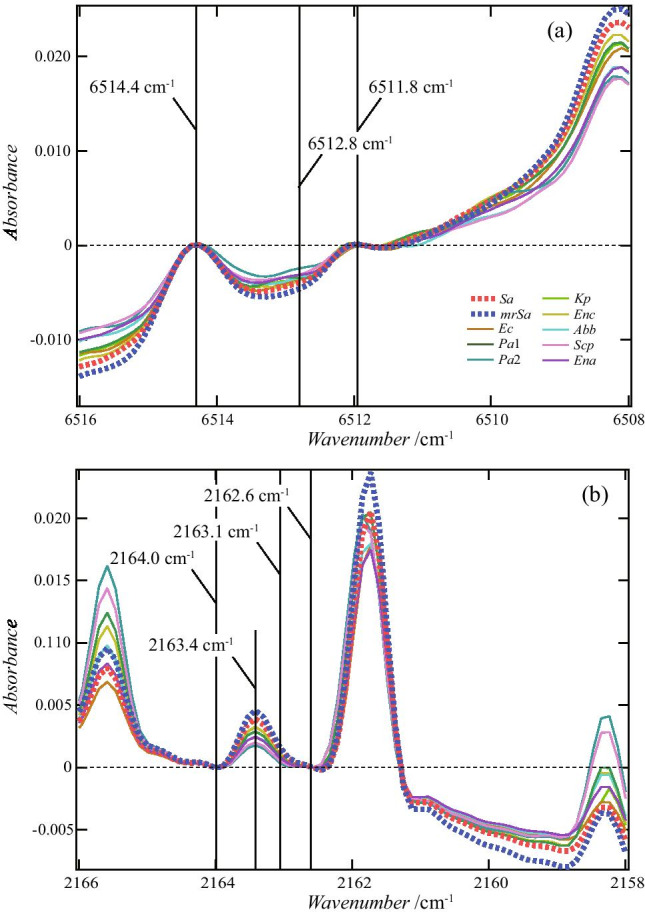
Relationship between 6514.4, 6512.8, 6511.8, 2164.0, 2163.1, and 2162.6 cm^−1^ selected in the decision tree. The values on the vertical axis were calculated such that the values of absorbance at the underlined wavenumbers in Fig. 9 became zero. Therefore, the values on the vertical axis of 6512.8 and 2163.1 cm^−1^ correspond to the values of the difference in absorbance used when creating the training data. As shown in **a**, because the absorbance values of 6514.4 and 6511.8 cm^−1^ were larger than the absorbance value of 6512.8 cm^−1^, the difference was negative, and the boundary value obtained by the decision tree was a negative value, − 3.189

The boundary value for this indicator is − 3.189. Figure 10 depicts the distribution obtained by this method. Figure 11 shows the wavenumbers used to create the decision tree. 6514.4 and 6511.8 cm^−1^ are the wavenumbers that give the peak absorbance, and 6513.2 cm^−1^ is the wavenumber near the bottom. It can be seen that the classification was performed using the absorbance of the two peaks on both sides of 6513.2 cm^−1^. By contrast, it can be seen that the wavenumbers of 2164.0, 2163.1, and 2162.6 cm^−1^ were selected to emphasize the absorbance change near the peak with 2163.4 cm^−1^.

### Relationship between infrared absorption spectrum and volatile organic compounds

Mass spectrometer studies have shown that *S. aureus* releases isovaleric acid and 2-methyl-butanal [9]. Therefore, the infrared absorption spectra of the two volatile organic compounds and the spectra of the gas around the *S. aureus* were compared. Isovaleric acid and 2-methyl-butanal were purchased from Tokyo Chemical Industry at purities > 99.0% (GC) and > 95.0% (GC), respectively. After filling the PA bag with nitrogen (purity > 99.99995%), each reagent was injected into the bag using a pipette. In addition, a sample containing pure water (filtered with Direct-Q 3UV, Merck Millipore S.A.S.) in a bag containing each reagent was also prepared. At the time of measurement by Fourier transform infrared (FTIR), the water vapor in the bag was saturated because the water remained as droplets on the inside surface of the bag.

**Fig. 12:**
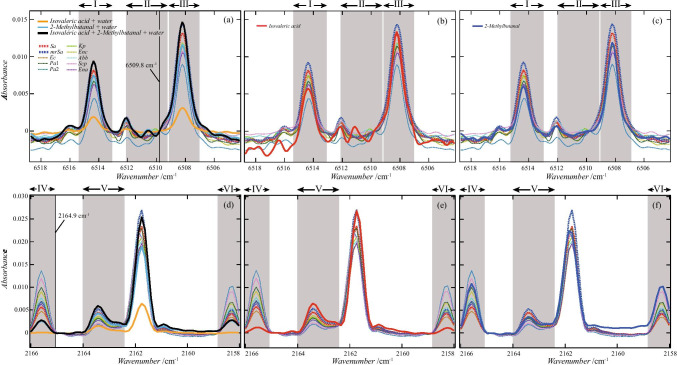
Absorbance curves of isovaleric acid, 2-methyl-butanoic, a mixture of isovaleric acid and water, and a mixture of 2-methyl-butanoic and water. Three thick solid curves are drawn in **a**. The three curves are the absorbance curve of isovaleric acid and water, that of 2-methyl-butanal and water, and that obtained by combining their two curves. The red dotted curve is the average value of absorbance of Sa, and the blue dotted curve is that of mrSa. The red solid line in **b** is the absorbance of isovaleric acid, and the blue solid line in **c** is the absorbance of 2-methyl-butanoic

The spectra of the gas around the bacteria were fitted with the spectrum of VOCs. The fitting curves are shown in Fig. 12. The spectra of isovaleric acid and 2-methyl-butanal are displayed superimposed on the spectra of the gas around the bacteria. The absorbances of isovaleric acid and 2-methyl-butanal were each multiplied by a certain magnification. The magnifications were determined by the least-squares method using the absorbances of *Sa* and *mrSa*. In regions I, III, and V, the wavenumbers that give peaks in the spectra of *Sa* and *mrSa* and the wavenumbers that give peaks in the spectra of isovaleric acid and 2-methyl-butanal were the same. However, regarding the absorbance of region II, the absorbance of the reagent was higher than that of *Sa* and *mrSa*. In addition, in regions IV and VI, the absorbance of isovaleric acid was low, and the absorbance of 2-methyl-butanal was higher than that of *Sa* and *mrSa*. The black solid lines in Fig. 10 (a) and (d) are curves obtained by adding the spectra of the mixed gas of isovaleric acid and water and that of the mixed gas of 2-methyl-butanal and water. The absorbance of isovaleric acid and water and the absorbance of 2-methyl-butanal and water were multiplied by different magnifications. All coefficients were calculated by the least-squares method. The shape of the black curve (isovaleric acid + 2-methyl-butanal + water) was closer to the shape of the spectrum of *Sa* and *mrSa* than the shape of the red curves (isovaleric acid) and blue curves (2-methyl-butanal).

From the above, the following can be considered.

1. In the mixed gas around bacteria, it is highly possible that the VOCs detected by a mass spectrometer do not exist alone, but are bound to other molecules, including water.
2. By synthesizing the spectra of isovaleric acid and 2-methyl-butanal, each containing water, it was possible to create a curve with shapes close to the spectra of *Sa* and mrSa. Therefore, the partial pressure of both VOCs is thought to affect the absorbance in the wavenumber region extracted by machine learning.
3. The black line did not completely reproduce the *Sa* and *mrSa* spectra of regions II, IV, and V. In particular, region II is an important region for distinguishing *S. aureus* from other bacteria. We surmise it could not be reproduced because many molecules other than isovaleric acid, 2-methyl-butanoic, and water were present in the mixed gas, and the molecules influenced each other. In other words, it can be said that it is impossible to distinguish bacteria by a deductive method that predicts the spectrum of a mixed gas by superimposing the infrared absorption spectra of molecules detected by a mass spectrometer. Therefore, an inductive method for measuring the spectrum of an actual sample and searching for a characteristic wavenumber using machine learning is necessary for the classification of mixed gases.

## Conclusions

This paper presented an approach for analyzing the infrared absorption spectra of gases surrounding bacteria using a decision tree–based machine learning algorithm. The proposed method offers an effective means to determine the presence (or absence) of the *S. aureus* bacteria. The wavenumber corresponding to the characteristic absorbance value of a spectrum can be determined using the decision tree algorithm. In this study, spectral classification was performed considering the differences between absorbance values corresponding to two or three points in the infrared absorption spectra. These differences were calculated using the following methods.

1. Considering the peak absorbance value and corresponding minima on either side of this peak. The baseline (minimum) values corresponding to the two adjacent points were subtracted from the peak value to determine the peak intensity.
2. Considering the absorbance values at two points (corresponding to fixed wavenumbers) and calculating the difference between them.
3. Considering the absorbance values at three points with constant wavenumbers, and of these points, we calculated a straight line connecting the two points on both sides. Subsequently, a vertical line was drawn from the remaining points to a straight line, and the difference in absorbance was calculated on the vertical line.

The results of this study reveal that all three methods are equally capable of identifying the gas produced by *S. aureus*. Thus, this study is the first of its kind to confirm the feasibility of using infrared adsorption spectra to measure and monitor the growth of *S. aureus.* The findings of this study are expected to afford humanity the realization of several health benefits arising from the use of such technologies in medical practice.
